# A Systematic Review of Methods for Increasing Vegetable Consumption in Early Childhood

**DOI:** 10.1007/s13668-017-0202-1

**Published:** 2017-04-29

**Authors:** Clare E. Holley, Claire Farrow, Emma Haycraft

**Affiliations:** 1 School of Sport, Exercise and Health Sciences, Loughborough University, Loughborough, LE11 3TU UK; 2 School of Health & Life Sciences, Aston University, Aston Triangle, Birmingham, B4 7ET UK

**Keywords:** Child, Repeated exposure, Fussy eaters, Peer modelling, Non-food reward, Bitter sensitive, Intervention duration

## Abstract

**Purpose of Review:**

This study aims to synthesise the body of research investigating methods for increasing vegetable consumption in 2- to 5-year-old children, while offering advice for practitioners.

**Recent Findings:**

Repeated exposure is a well-supported method for increasing vegetable consumption in early childhood and may be enhanced with the inclusion of non-food rewards to incentivise tasting. Peer models appear particularly effective for increasing 2–5-year-olds’ vegetable consumption. There is little evidence for the effectiveness of food adaptations (e.g. flavour-nutrient learning) for increasing general vegetable intake among this age group, although they show some promise with bitter vegetables.

**Summary:**

This review suggests that practitioners may want to focus their advice to parents around strategies such as repeated exposure, as well as the potential benefits of modelling and incentivising tasting with non-food rewards. Intervention duration varies greatly, and considerations need to be made for how this impacts on success.

## Introduction

The prevalence of paediatric obesity has increased significantly in recent years, with more than 40 million children under the age of 5 overweight or obese globally [1], and one in five reception-age children in the UK measuring as overweight or obese [2]. Concurrently, vegetable consumption in both adults and children falls below recommended levels in the UK [3], with less than one in five pre-schoolers consuming five portions of fruit or vegetables a day [4, 5].

Diets rich in fruits and vegetables can not only lower children’s caloric intake and reduce the risk of obesity [6] but can also serve to prevent against many non-communicable diseases [7–10]. For example, raw and leafy green vegetables are thought to be particularly protective against cardiac events [11]. Meanwhile, recent evidence from the World Health Organisation asserts that low fruit and vegetable intake is one of the five leading behavioural and dietary risk factors for cancer development [12].

Although research implicates both fruits and vegetables in disease protection and prevention, it is known that whilst increasing fruit consumption has health benefits, fruits are also high in naturally occurring sugars. As a calorific compound, excess sugar consumption is a major contributor towards overweight and obesity [13], as well as type two diabetes. Furthermore, some research suggests that fructose consumption activates the digestive system in a different way to glucose, so that it does not stimulate insulin or leptin release [14]. This in turn can result in weight gain and an increased risk of diabetes. However, according to parental reports, 46% of foods disliked by children are vegetables, while just 8% are fruits [15]. With this in mind, efforts to increase vegetable consumption are needed and should be a focus for both researchers and practitioners.

Given the health benefits of eating fruits and vegetables, and the fact that eating behaviours track across childhood and into adulthood [16, 17], it is important to establish a healthy level of intake of these foods early in life. With particularly low levels of vegetable intake in many young children [4, 5], it is important to focus on methods to increase vegetable consumption early in childhood, where the maximal benefits of a diet rich in vegetables can then be experienced across the lifespan.

Previous research has investigated a number of possible methods for increasing children’s intake of disliked foods, with most of this research focusing on the notion that children need to be exposed to new and disliked foods numerous times in order for them to become liked and accepted [18, 19]. A large number of studies have investigated the possible methods which can be used alongside repeated exposure to encourage children’s consumption of rejected foods, including using non-food rewards [20–22], parental modelling [23, 24], teacher modelling [25], peer modelling [26–28], flavour-flavour learning [19, 29, 30] and nutrient learning [31]. With a plethora of studies in the broad area of improving children’s consumption of foods, it is necessary to synthesise the relevant literature relating to young children’s consumption of vegetables. This will allow researchers to identify areas which require further investigation, whilst helping to ensure that practitioners and experts in the field have a clear view of the evidence base.

In summary, research suggests that vegetable consumption can provide a wide range of health benefits and seems to protect against a number of diseases. Furthermore, vegetables in particular have been shown to have positive effects on health outcomes, such as cardiac health, but are often poorly consumed by young children. To our knowledge, only two previous systematic reviews have been conducted on increasing children’s vegetable intake, but these have focused on a broader age range of children, and on interventions which tackle both fruit and vegetable consumption [32, 33]. The current systematic review therefore aims to fill a gap in the extant knowledgebase and focuses on the possible methods for increasing vegetable consumption in children aged 2 to 5 years.

## Methods

### Search Strategy

An online literature search was conducted using the search engines Web of Science and PubMed. Key terms relating to children’s consumption of vegetables were used to identify potentially relevant papers for this review. Key terms included child vegetable consumption, intervention, modelling, reward, flavour-flavour, flavour-nutrient, repeated exposure, messy play and tactile play. These search terms were utilised in various combinations, using the operator AND. Relevant articles were extracted up until November 2016.

### Definition of Terms

Key terms were identified from the authors’ knowledge of the literature, some of which may require definition. One such key term was flavour-flavour learning, which is a method of learning to like a food by trying it repeatedly when paired with another food or flavour which is already liked [29]. Another key term used in this review is flavour-nutrient learning. Flavour-nutrient learning is the process by which a flavour becomes liked and accepted because it is associated with high nutrient content. A further key term is the method of modelling. Modelling is the process by which an individual or group of individuals demonstrates a behaviour which they would like another individual to perform and can be thought of as leading by example. This method is grounded in Social Learning Theory, which states that behaviour is learned by observation [34]. The last key term to be defined is reward. In the context of this review (and indeed the literature), a reward is defined as something which is given in exchange for a desired behaviour, which in the case of this literature is the tasting or consumption of vegetables. Rewards are described to the individual before a request for the behaviour is made (they are incentives), and the reward is only given if this behaviour is performed (they are contingent).

### Inclusion and Exclusion Criteria

To be eligible for inclusion in this review, articles were required to have increasing young children’s vegetable consumption as one of their main aims, children’s consumption of vegetables as the main outcome variable and to be published in a peer-reviewed journal. Studies were also required to have an experimental design, where clear cause and effect of methods implemented could be ascertained. Papers were excluded if the children were younger than 2 or older than 5 years, if they did not target the general population (e.g. if they specifically targeted a clinical group or those with low consumption) or if the statistics for vegetable consumption alone were absent. Papers were also excluded if they were not published in English or if they employed a cross-sectional design rather than an experimental design.

### Identification of Appropriate Articles

**Fig. 1:**
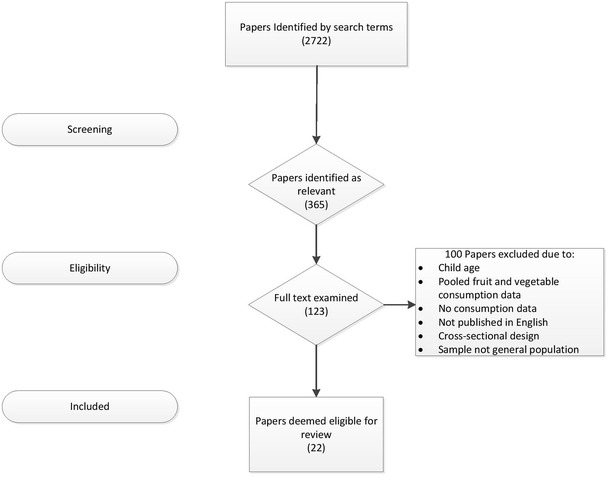
Flow diagram of identification process for papers included in this systematic review

Initially, relevant papers were identified by screening the article titles. The abstract of relevant articles was then read to check that the paper aligned with the required inclusion and exclusion criteria. Those papers whose abstract either met these criteria or failed to adequately describe the details of these criteria were then downloaded and read in full (Fig. 1).

### Data Extraction

Data were extracted from the included articles by the first author, on a standardised form developed for this review. Extracted data included author(s), date of publication, country of study, study aim, sample characteristics (sample size, age and other defining characteristics), the main method of increasing vegetable consumption being tested, design, methods, intervention duration, outcome measures, findings and take-home message. A summary of this information can be found in Table 1.

**Table 1:** Summary of extracted data from papers included in a systematic review of methods used to increase 2- to 5-year-olds’ consumption of vegetables

Authors	Year	Sample	Country/setting	Main method(s) used to encourage vegetable consumption	Design	Intervention duration	Findings	Take-home messages
Bouhlal et al. [35]	2014	151 2–3-year-olds	France/nursery	Repeated exposure (RE) Flavour-flavour learning (FFL)	Between-subjects	8 exposures	Significantly increased liking and acceptance of a relatively novel and neutrally liked vegetable in all groups. Post-intervention increases highest among the RE group. Increases in consumption (mean = 64 ± 11 g) maintained at 6 months	RE is a simple and effective method to increase vegetable intake in the short (5 weeks) and long term (6 months) in toddlers. No evidence of FFL
Capaldi-Phillips et al. [36]	2014	29 3–5-year-olds	USA/preschool	RE FFL/flavour-nutrient learning (FNL) (associative conditioning)	Between-subjects	7 exposures	Pairing tastings of Brussels sprouts with cream cheese (FFL) significantly increased children’s liking of Brussels sprouts regardless of type (FNL), and children who liked the sprouts more consumed significantly more (mean = 10.79 g) than children who disliked the sprouts (mean = 1.76 g). No difference in liking or consumption of cauliflower between groups (FFL, FNL or RE)	Associative conditioning with cream cheese (FFL) can significantly increase liking of bitter vegetables. RE was not effective at improving liking for bitter vegetables (Brussels sprouts) but it was for cauliflower
Correia et al. [37]	2014	57 children (mean age 4.4 years)	USA/preschool	Visual appeal FFL	Cluster randomised crossover design	Single session	Neither visual appeal enhancement nor pairing with a liked food (FFL) increased vegetable consumption. Pairing increased willingness to try the vegetable (defined as consumption of 3 g or more) from 79 to 95% of children	Pairing of a vegetable with a familiar food (FFL) may increase children’s willingness to try a vegetable
de Wild et al. [31]	2013	28 2–4-year-olds	Netherlands/preschool	FNL RE	Crossover intervention	7 weekly exposures	Significant increase in children’s consumption of a relatively novel and disliked vegetable soup post-intervention (mean = 58 g), 2- and 6-month follow-up. No effect of energy content of soup on children’s consumption (FNL). High energy paired flavour of soup liked significantly more post-intervention, but this did not remain significant at 2-month follow-up	No evidence for FNL in consumption but tentative evidence for the effectiveness of FNL for increasing preference in the short term. Robust evidence of a repeated-exposure effect
de Wild et al. [38]	2015	39 1.5–4-year-olds	Netherlands/nursery	FFL RE	Crossover intervention	7 weekly exposures	Significant increase in consumption of relatively novel beetroot and parsnip crisps post-intervention (mean = 7–9 g), regardless of whether paired with familiar and liked ketchup or white sauce No significant effect of FFL Findings maintained at 2 and 6 months	RE is the primary mechanism for increasing vegetable consumption in young children, with evidence of long-term effects
de Wild, et al. [39]	2015	70 2–4-year-olds	Netherlands/home	Providing a choice of vegetables	Randomly assigned between subjects	12 occasions (6 different vegetables)	Significant but not robust model found to predict vegetable consumption including whether children were offered a choice, baseline liking, child age and gender. Whether children were offered a choice was not a significant predictor	Marginal but not robust effect of choice of vegetable on consumption. Effect of choice influenced by numerous other factors (e.g. age, liking)
Fildes et al. [40•]	2013	221 pairs of twin 3-year-olds	UK/home	Non-food rewards	Randomised controlled trial	14 daily exposures	Intake and liking of the target vegetable increased significantly more in children who experienced daily tastings of a disliked vegetable paired with stickers (mean = 4.07 ± 7.52 g) than control children (no treatment; mean = 0.61 g ±4.35)	Parent-administered interventions utilising non-food rewards can be effective for increasing children’s consumption of vegetables and do not require professional contact
Fisher et al. [41]	2012	152 3–5-year-olds	USA/preschool	FFL FNL	Between-subject design	13 daily exposures	No main effect of dip condition (FNL or FFL). But dip × bitter sensitivity interaction. Bitter-sensitive children ate 80% more of the bitter vegetable with dip or sauce (mean = 14.7 g) than vegetable served plain (mean = 8.1 g)	Dip/sauce (FFL) may be a successful method to increase consumption of brassicas among bitter-sensitive children
Gripshover et al. [42••]	2013	40 4–5-year-olds	USA/preschool	Nutrition education	Cluster randomised controlled trial	Maximum of 2 sessions per week for 12 weeks	Intervention children increased their consumption by significantly more pieces of vegetable (mean = 6.15 pieces) than control children (mean = 2.08 pieces)	Intuitive-theory-based nutrition education interventions could be effective in helping children eat healthier foods
Harnack et al. [43]	2012	53 2–5-year-olds	USA/preschool	Serving first—before the rest of the main meal	Randomised crossover experiment	10 daily sessions	No significant difference in vegetable intake when served in advance of the rest of meal compared to when served as part of a main meal for 2 weeks	Serving vegetables first does not appear to increase vegetable intake
Hausner et al. [44]	2012	104 2–3-year-olds	Denmark/nursery	FFL FNL RE	Non-randomised between subjects	10 exposures	Significantly increased consumption of a novel vegetable in both the FFL (5.8× baseline consumption) and RE (4.6× baseline consumption) groups after, maintained at 6 months post. No significant effect of FNL	RE effective for increasing vegetable consumption. Highly neophobic children (those with high levels of fear of new foods) may benefit from pairing vegetables with a known and liked flavour (FFL)
Holley et al. [45•]	2014	115 2–4-year-olds	UK/home	RE modelling non-food rewards	Non-randomised controlled trial	14 daily exposures	Significant increased consumption of a disliked vegetable in the modelling, non-food rewards and RE group (mean = 3.96 g) and in the non-food rewards and RE group (mean = 3.65 g), compared to the control group (mean = 1.14 g). Significant differences in liking of target disliked vegetable post-intervention, with liking highest in the modelling, non-food rewards and RE group	Parent-led interventions utilising non-food rewards and modelling alongside RE may be cost efficient for increasing children’s vegetable consumption
Horne et al. [46]	2011	20 children aged 24–52 months	UK/preschool	Mixed methods (modelling, non-food rewards)	Within subjects	30 days	Threefold increase in target vegetable consumption post-intervention (from 28.8 to 85.5% of ∼25 g portion), maintained at 6-month follow-up	Peer modelling and non-food rewards intervention successful in a preschool setting
Remington et al. [21]	2012	173 3–4-year-olds	UK/home	RE non-food rewards	Randomised controlled trial	12 daily exposures	Tangible non-food rewards increased children’s intake and liking of the disliked target vegetable significantly more than controls (mean difference = 1.27), maintained at 3-month follow-up. Social reward group not significantly different to control	Support for parental use of tangible non-food rewards with repeated taste exposures to improve children’s vegetable consumption
Roe et al. [47]	2013	61 3–5-year-olds	USA/nursery	Serving a variety of vegetables at once	Crossover design	4 sessions	Serving a variety of 3 vegetables significantly increased vegetable consumption (mean = 22 ± 1 g) compared to serving any of the vegetables individually	Serving a variety of vegetables as a snack could help preschool children meet recommended intakes
Spill et al. [48]	2011	72 3–5-year-olds	USA/nursery	Portion sizes	Crossover design	Single session	Increasing the portion size increased soup and vegetable intake. Largest portion (300 g soup, larger than an average serving for this age group) resulted in the highest consumption of soup (mean Δ = 25 g), while 150 g portion of soup (smaller than an average potion for this age group) resulted in significantly reduced intake of the concurrent main course	Serving low-energy-dense, vegetable soup as a first course is an effective strategy to reduce children’s intake of a concurrent main meal and increase vegetable consumption
Staiano, et al. [49]	2016	42 3–5-year-olds	USA/preschool	Modelling	Randomised controlled trial	Single session	Children who watched a video of a peer modelling vegetable consumption ate significantly more vegetables (mean = 15.5 g) and demonstrated higher preference for eating them again than two control groups (non-vegetable related video or no video; mean = 5.9 g) 7 days post-intervention, with no differences on post-intervention or 1 day post-intervention	Screen-based peer modelling is a promising tool to improve children’s vegetable consumption
Witt et al. [50]	2012	122 children (majority aged 4–5, no detail)	USA/nursery	Mixed methods (songs about vegetables, looking, touching and tasting)	Cluster randomised controlled trial	6 weeks	‘Colour Me Healthy’ programme significantly increased consumption of vegetable snack (mean Δ = 33.1%). No increase seen in control group	Childcare centres could be useful outlets for interventions. Mixed-methods interventions with various sensory activities seem effective at increasing vegetable consumption
Wolfenden et al. [51]	2014	328 3–5-year-olds	Australia/home	Mixed methods (increasing availability, parent modelling, family mealtimes)	Cluster randomised controlled trial	4 weeks	Significantly higher child vegetable intake at 12 months’ follow-up among intervention group (Children’s Dietary Questionnaire score mean = 2.95 ± 0.12) than control (mean = 2.47 ± 0.11). Increased consumption not maintained at 18 months	Telephone-based interventions seem effective, although these may need maintenance to promote long-term vegetable consumption

## Results

The oldest papers included in the review were published in 2011, with the newest published in 2016. Multiple papers tested a number of methods for increasing children’s consumption of vegetables, including repeated exposure, flavour-flavour learning, flavour-nutrient learning, associative conditioning, rewards, modelling and visual aspects (e.g. presenting vegetables arranged in the shape of a caterpillar). Individual papers also explored additional methods such as portion size, variety, nutrition education, serving vegetables first and serving vegetables with dips. In order to facilitate discussion of study findings in this review, these methods were grouped according to common themes or were assigned their own section if methods were in stark contrast to those described in other sections. These themes were (1) repeated exposure, (2) food adaptations (including flavour-flavour learning, flavour-nutrient learning and visual presentation), (3) mealtime adaptations (such as serving vegetables first, serving larger portions of vegetables and providing a choice of vegetables), (4) social factors (including modelling and non-food rewards), (5) nutrition education and (6) mixed methods. Summaries of the findings, grouped according to these themes, appear below.

### Repeated Exposure

Seven papers included in this review explored the effectiveness of repeated exposure alone for increasing children’s liking and consumption of vegetables [21, 31, 35, 36, 38, 44, 45]. With this method, children are repeatedly offered and encouraged to try a target vegetable, with papers in this review testing 7 to 14 exposures. In line with research among older children [22, 52, 53], all but one [36•] of these studies found good evidence for repeated exposure as an effective tool for increasing pre-school children’s consumption of vegetables, including at 6-month follow-up. In this one inconsistent study from Capaldi-Phillips et al. [36•], repeated exposure was effective for cauliflower liking but not for Brussels sprouts. In this study, the only measure of ‘success’ of repeated exposure was the proportion of children who liked or consumed the target vegetables after the 14-day conditioning period, where it is possible that repeated exposure may have increased the children’s consumption, but change in consumption was not measured. Moreover, Capaldi-Phillips et al. [36•] posit that repeated exposure has not previously been tested with extremely bitter vegetables as they did and may not be effective enough to transform consumption of these. This idea requires further testing with baseline and post-intervention data, as well as comparison with a control group. It should also be noted that many of the papers in this review have tested the ability of repeated exposure combined with other methods to increase young children’s vegetable consumption. In this way, repeated exposure can be considered the central method being tested in this review.

### Food Adaptations

The largest proportion of papers (*n* = 9) captured in this review refers to making adaptations to vegetables in order to increase children’s consumption of these foods. These methods typically took one of three forms: flavour-flavour learning, flavour-nutrient learning and visual presentation. Six of these papers tested the effectiveness of flavour-flavour learning to increase children’s consumption of vegetables [35, 36•, 37, 38, 41, 44]. While most of these studies found no advantage of flavour-flavour learning beyond that of repeated exposure [35, 37, 38, 44], one study [41] suggests that offering familiar and liked dips to children who are sensitive to bitter tastes may be useful for increasing consumption of brassicae (green, cruciferous vegetables) in the short term when served with dip. Further to this, the research suggests pairing bitter vegetables (Brussels sprouts) with cream cheese (with or without added sugar) for 14 days can significantly increase children’s rated liking of Brussels sprouts when subsequently presented plain, although this did not increase their consumption [36•]. However, children who reported greater liking for the Brussels sprouts also consumed more of the sprouts than children who reported disliking the sprouts, which can be seen as an indirect effect on children’s consumption of the vegetable.

Four papers included in this review examined the possible utility of flavour-nutrient learning for increasing children’s consumption of vegetables [31, 36•, 41, 44]. However, none of these papers found a significant effect of flavour-nutrient learning on pre-school children’s consumption of vegetables. One of these studies found that adding energy in the form of oil to vegetable soup (which was hypothesised to cause flavour-nutrient learning) had no effect on children’s consumption of soup, although there was a significant short-term increase in liking for the soup with high energy content [31]. Moreover, Hausner et al. [44] found that adding oil to artichoke puree did not increase children’s consumption of the puree. Capaldi-Phillips et al. [36•] found that although serving with cream cheese could increase children’s liking of Brussels sprouts, and that children who liked the sprouts more consumed more when later served sprouts alone, this effect was not boosted when cream cheese was served with added sugar (and so had higher energy content). Finally, Fisher et al. [41] found that whilst serving broccoli with ranch dressing dip could increase bitter-sensitive children’s broccoli consumption, there was no difference in this effect when the energy content was manipulated.

Correia et al. [37] examined whether presenting vegetables in a visually appealing way could increase children’s consumption of vegetables. Here, cucumber was served either arranged in the shape of a caterpillar or simply served on a plate. The authors found that presenting cucumber in a visually appealing way did not increase children’s consumption of the cucumber.

### Mealtime Adaptations

Four papers captured in this review referred to mealtime adaptations as possible methods for increasing vegetable consumption in early childhood. These ranged from serving vegetables first, to providing a choice, or serving a selection of vegetables, and also serving larger portions of vegetables. Harnack et al. [43] investigated whether serving vegetables 5 min before the rest of a meal could be an effective method for increasing vegetable consumption, but failed to find a significant effect of doing so. A study by de Wild et al. [39] examined whether giving young children a choice of vegetables could increase their concurrent consumption of vegetables. Children in the experimental group were given a choice between two vegetables at mealtimes for 12 days, while control group children were simply served a vegetable with each meal. The authors reported a significant but not robust effect of choice on children’s consumption. Here, choice was not a significant predictor of children’s consumption of vegetables. Roe et al. [47] compared children’s consumption of vegetables as a snack when serving a variety of vegetables, as opposed to being served any of the chosen vegetables individually. Serving a variety of vegetables was found to increase children’s consumption of vegetables. The last study to investigate the utility of mealtime adaptations tested whether serving larger portions of vegetables to young children could increase their consumption [48]. In this study, children were served a small, medium or large portion of tomato soup before their main meal, or no soup. It was found that increasing the portion of soup served at the beginning of the meal increased children’s consumption of the soup, and so their intake of vegetables.

### Social Factors

Six papers explored the potential for social influences such as modelling and non-food rewards to increase pre-school children’s consumption of vegetables. Four papers examined the effects of non-food rewards [21, 40•, 45•, 46]. These papers unanimously found that pairing repeated exposure with rewards in the form of stickers was a successful method for increasing children’s liking and consumption of disliked vegetables.

Two papers looked at the effectiveness of modelling to increase children’s consumption of vegetables. Holley et al. [45•] examined whether caregivers trying a target disliked vegetable in front of their child before offering the vegetable to their child to try could increase their child’s willingness to try the vegetable, and consequently their consumption and liking of that vegetable. They found that offerings paired with modelling were not effective at increasing children’s liking or consumption of the target vegetable, but that offerings paired with both modelling and non-food rewards were. Staiano et al. [49] investigated whether viewing a video of a peer model consuming a vegetable could increase pre-schoolers’ consumption of that vegetable. It was found that children consumed significantly more of the target vegetable after viewing the peer modelling video than after viewing a similar video segment unrelated to food consumption.

### Nutrition Education

Just one study [42••] investigated the impact of nutrition education at improving young children’s consumption of vegetables. In this study, children were educated on the nutritional importance of vegetables in general, including being read nutrition education books twice per week for a period of approximately 10 weeks. Children who had experienced this nutrition education significantly increased their vegetable consumption and ate more pieces of vegetables (mean = 6.15 pieces) than control children (mean = 2.08 pieces) during a post-intervention free-choice snack session in which a variety of foods were served which was identical to a pre-intervention session.

### Mixed-Methods Interventions

Three papers explored the effect of mixed-methods interventions (i.e. where two or more methods were combined within experimental conditions) on children’s vegetable consumption [46, 50, 51]. These three papers used starkly contrasting methods. In the first, Horne and colleagues [46] examined the utility of an intervention containing both peer modelling and non-food reward components for increasing children’s consumption of vegetables. Children were shown videos of fictional cartoon peers consuming vegetables, as well as being read letters from the peers urging them to consume vegetables to help them save the world. Children who tasted and consumed vegetables were then rewarded with small rewards such as stickers, which accumulated resulted in larger non-food rewards. Children who participated in the intervention demonstrated significant increases in consumption of both vegetables specifically targeted by the intervention (from 28.8 to 85.5% of ∼25 g portion) as well as other vegetables (from 40.2 to 58.9% of a ∼25 g portion), and these increases were maintained at 6-month follow-up.

In the second mixed-methods paper, Wolfenden et al. [51] tested a 4-week intervention involving increasing availability of vegetables in the home and parental modelling of vegetable consumption, and supporting family mealtimes for its ability to increase pre-school children’s consumption of vegetables. It was found that children who participated in the intervention had significantly higher vegetable consumption at 12-month follow-up as measured by the Children’s Dietary Questionnaire [54], although this effect was not seen at 18-month follow-up.

The final mixed-methods paper in this review, from Witt et al. [50], investigated the efficacy of a 6-week education programme (*colour me healthy*) including songs about vegetables, looking at, touching and tasting vegetables for increasing children’s consumption of vegetables. Children in the intervention group significantly increased their consumption of vegetables 3 months post-intervention, although owing to the design of this study, it is unclear which method(s) this increase in consumption can be attributed to.

## Discussion

This paper systematically reviewed and evaluated experimental research which aimed to increase vegetable consumption in children aged 2 to 5 years. The overall aim of this review was to assess the possible methods for increasing vegetable consumption in early childhood and to explore how effective these methods are. A total of 22 papers were included in the review, and these investigated a number of methods which could be grouped into common themes: repeated exposure, food adaptations, mealtime adaptations, social factors, nutrition education and mixed methods.

One well-documented method for increasing young children’s vegetable consumption is repeated exposure. All but one of the papers in this review which examined repeated exposure found it to be effective for increasing children’s consumption [21, 31, 35, 38, 44, 45]. These interventions which showed positive effects ranged from 7 to 14 days in duration. While previous research suggests that 15 exposures might be necessary to achieve acceptance of novel foods among 3- to 4-year-olds [19], this review suggests that seven exposures may in fact be sufficient for some children, at least in the short term. Further research is needed to establish whether this is long enough for promoting long-term vegetable acceptance and whether this depends on factors like temperament. It may be the case that while only 7 exposures are necessary to increase consumption in the short term, or in some children, 14 exposures are more beneficial for sustained increases in consumption, as shown by Remington and colleagues [55]. Capaldi-Phillips et al.’s [36•] study which failed to corroborate these positive results did not measure consumption change, and so it is possible that the repeated exposure group of children’s consumption did increase. Overall, this review supports the notion that repeated exposure is highly important for increasing children’s vegetable consumption.

This review highlighted that considerable research has been conducted into the possible utility of food adaptations for increasing consumption of vegetables among this young age group. However, the vast majority of this research has proven unsuccessful, with many studies failing to find support for flavour-flavour learning [35, 37, 38, 44] or flavour-nutrient learning [31, 36•]. That being said, there is some limited research which suggests that flavour-flavour learning may be a useful method for increasing young children’s liking and intake of bitter tasting vegetables, particularly among children who have higher sensitivity to this flavour dimension [34, 44]. With this in mind, flavour-flavour learning warrants further research, including long-term follow-up, specifically in relation to bitter vegetables to which many children have a natural aversion [56]. In summary, pairing disliked vegetables with liked flavours may be a useful method for increasing vegetable consumption in especially fussy children or with more bitter vegetables, but based on this review, it does not seem to warrant being recommended as a primary method of increasing vegetable consumption in children aged from 2 to 5 years.

A number of papers examined whether mealtime adaptations could improve children’s consumption of vegetables. While neither serving vegetables in advance of the rest of the meal nor offering children a choice of vegetables increased their consumption [36•, 52], serving children a variety of vegetables [47] and serving a large portion of vegetable soup as a first course to a meal was successful at increasing children’s intake of vegetables [48]. Although an interesting finding, serving an additional course at mealtimes may not be a feasible method for many parents, particularly given that the burden of preparation time and cost are commonly stated barriers to increasing vegetable consumption [57–59]. Moreover, it is possible that this method might result in parents offering too much food, which may have a negative impact on child weight. In support of this notion, previous research asserts that when served a larger portion adults and children eat more [60, 61], so parents should be cautious of increasing portion sizes of calorific foods. Overall, this research suggests that mealtime adaptations may not be the most useful methods for increasing young children’s vegetable consumption.

Social methods, including non-food rewards and modelling, have also been researched for their ability to increase children’s consumption of vegetables. All of the papers which investigated the utility of non-food rewards included in this review found that they were indeed effective for increasing pre-school children’s consumption of vegetables [21, 40•, 45•, 46], with evidence for results being maintained at 3-month [21] and 6-month follow-up [46]. Therefore, this review strongly supports the use of non-food rewards to incentivise children’s consumption of vegetables.

Modelling was another social method which was explored in this review. Here, findings were mixed, with one study indicating that parental modelling alone may not be sufficient to increase consumption but that it might be effective alongside other factors [45•], while another suggests that peer models may be effective [49]. Moreover, Horne et al.’s [46] mixed methods studies indicate that peer modelling can be a component of successful mixed-methods interventions; meanwhile, in studies by both Holley et al. [31] and Witt et al. [50], children who participated in an intervention including parent models consumed significantly more vegetables. This research indicates that among 2- to 5-year-olds, if taken as a standalone method, peer models may be more effective for eliciting behaviour change than parental models. It is possible that as children of this age are just beginning to form friendships that the power of peer models is different, with previous research suggesting that among older children models which are more admired are more powerful [62]. However, mixed-methods interventions appear to benefit from the inclusion of role models—be they peer or parent—for promoting increased vegetable consumption in children. It is apparent that pre-school children generally spend a larger amount of time with their parents than older children, including more shared mealtimes, and this may well moderate the impact of parental modelling for this age group.

Just one paper which qualified for this review investigated the utility of nutrition education for increasing vegetable consumption among pre-schoolers [42••]. With multiple studies exploring the efficacy of this method for older children, and finding it to be effective [63, 64], it may be that pre-schoolers are perceived as not as capable of understanding nutrition education. However, the paper that tested the efficacy of this method for young children found that children did indeed increase their vegetable consumption [42••]. Moreover, Witt et al.’s [50] mixed-methods study, which educated children about vegetables through songs, significantly increased children’s vegetable consumption for 12 months. This suggests that nutrition education may be feasible for this pre-schooler age group, and further research should explore this. It should be acknowledged that the significant results in Witt et al.’s [50] mixed-methods study could also be attributed to the sensory education and exposure which the children received in the forms of songs, site, touch and taste. Here, literature with both a younger and a broader age range of children suggests that sensory exposure can be used to increase children’s willingness to taste vegetables [65, 66].

This paper presents a systematic review of experimental published papers which have increasing vegetable consumption in 2- to 5-year-old children as a primary aim. As such, it synthesises the evidence base, allowing an unbiased observation of the progress of the field as a whole. However, it is limited to strictly those studies with participants aged from 2 to 5 years. This necessitated the exclusion of several studies which investigate possible methods to use with children aged from 4 to 6 years as well as those younger than two. It is possible that these studies can also contribute knowledge for 2- to 5-year-olds, and this review is not intended as a standalone information source for the entire field. Moreover, we have focused on only studies with an experimental design, and so may have missed out on naturalistic evidence to support methods for increasing children’s vegetable consumption. Furthermore, there are clear gaps in the knowledgebase of the field. For example, there is limited research into the long-term efficacy of many of the methods presented, with just one quarter of the studies included in this review reporting even a 6-month follow-up. Moreover, there is a precedent for unblinded trials in the field, where possible experimenter effects are not adequately discussed. Additionally, while some studies in this review demonstrate that a single session can result in changes in children’s consumption of vegetables [48, 49, 67], further evidence is needed to demonstrate that such methods can continue to work across multiple sessions, rather than demonstrating a novelty effect. Finally, it should be considered that although only findings pertaining to vegetables were included in this review, some of the studies did also seek to increase fruit consumption. It is possible that the mere presence of fruit in these studies may have had a detrimental effect on children’s consumption of the study vegetables, diluting intervention effects which may have otherwise been seen. In order to assess this notion, it would be interesting to compare the effects found in studies which only tackle vegetable consumption with those which seek to increase intake of both fruit and vegetables.

In summary, the current review demonstrates that repeated exposure is likely the most successful method of increasing vegetable in early childhood. However, it is clear that in order for children to achieve the tastings necessary to acquire liking and acceptance of vegetables, other methods might be necessary alongside repeated exposure. The literature presented in this systematic review suggests that non-food rewards are likely to be a successful method for achieving these tastings, where (as also stated in a previous review of reward literature [56, 68]) the over-justification hypothesis once presented as an argument against using rewards now seems implausible. Modelling may also be an effective tool, and this review suggests that this may be most effective as part of mixed-methods interventions. In terms of food adaptations, these seem unlikely to be beneficial for the majority of children or indeed the majority of vegetables, although future research should explore whether pairing vegetables with liked flavours may be useful with either particularly bitter vegetables, or particularly bitter-sensitive or fussy children. Lastly, nutrition education is an under-researched avenue for this age group, where the scant evidence that there is seems favourable. Researchers should consider how nutrition education programmes might be implemented in a suitable way for pre-school children. Having said this, it should be acknowledged that it is highly possible that gaps in the literature presented here may be due to publication bias, where those studies which have failed to garner significant findings have not been published. In reference to this, efforts should be made to ensure that the platform for publication is on the basis of methodology rather than findings.

## Conclusion

Although vegetable consumption remains an area of concern for public health, more research is needed into which methods might be truly effective for increasing vegetable consumption in early childhood. Future research in this area should focus on (a) bitter vegetables most commonly rejected and (b) presenting longitudinal evidence of the efficacy of previously demonstrated methods. In conclusion, this review suggests that repeated exposure is a highly effective method for increasing children’s vegetable consumption which may benefit from being paired with modelling by peers or parents, as well as non-food rewards, with tentative evidence for the use of alternative methods which require further exploration.
